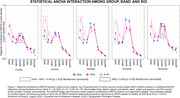# Abnormal cortical neural synchronization mechanisms in quiet wakefulness in patients with Symptomatic Huntington and Dementia due to Alzheimer’s disease

**DOI:** 10.1002/alz.090669

**Published:** 2025-01-03

**Authors:** Dharmendra Jakhar, Federico Tucci, Giuseppe Noce, Susanna Lopez, Roberta Lizio, Claudio Del Percio, Matteo Carpi, Enrico Michele Salamone, Bahar Güntekin, Görsev Yener, Marina De Tommaso, Marianna Delussi, Claudio Babiloni

**Affiliations:** ^1^ Sapienza University of Rome, Rome Italy; ^2^ IRCCS Synlab SDN, Naples Italy; ^3^ Istanbul Medipol University, Istanbul Turkey; ^4^ Izmir University of Economics, Faculty of Medicine, Balçova, Izmir Turkey; ^5^ University of Bari Aldo Moro, Bari Italy; ^6^ San Raffaele Cassino, Cassino Italy

## Abstract

**Background:**

Huntington’s disease (HD) is a progressive neurodegenerative disorder phenotypically manifested by motor, cognitive and psychiatric symptoms (Novak and Tabrizi, 2011). These patients are also characterized by vigilance abnormalities. This has been demonstrated by electrophysiological measures (Wiegand et al., 1991). In particular, previous studies have shown that HD patients have reduced alpha amplitude (de Tommaso et al., 2003; Bellotti et al., 2004) and increased resting delta activity (Bylsma et al., 1994). These electroencephalographic changes resemble those observed in patients with Alzheimer’s dementia (Babiloni et al., 2020). To date, there have been only a few studies on the specific pathophysiological changes that are the cause of the above changes in brain rhythms. In our opinion, the comparison of these two populations could be an enrichment of the physiological model that explains the electroencephalographic changes that have been observed in HD patients.

**Method:**

Clinical and EEG datasets in 21 ADD (Dementia due to Alzheimer Disease), 16 S‐HD (Symptomatic Huntington Disease) and 39 Healthy (Nold) individuals matched for demography, education, and gender were taken from an Eurasian database. The rsEEG frequency bands were individual delta, theta, alpha as well as fixed beta (14‐30 Hz) and gamma (30‐40 Hz). The eLORETA freeware was used to estimate cortical rsEEG sources at delta, theta, alpha, beta, and gamma frequency bands during eyes‐closed and‐open conditions.

**Result:**

As core findings, we have showed that global delta source activities (and central, parietal, and occipital delta source activity) are significantly greater in S‐HD than in ADD (p<0.05 Bonferroni corrected) and that frontal and temporal alpha 2 source activities are significantly lower in S‐HD than in ADD (p<0.05 Bonferroni corrected) Showed in Figure 1.

**Conclusion:**

The present findings showed more abnormalities in delta source activities in HD patients if compared to the ADD patients, possibly related to an abnormal functioning of dopaminergic ascending neuromodulation of basal ganglia in HD subjects. Moreover, the HD peculiar alterations in alpha source activities could be related to more alterations in the cholinergic system impinging upon basal ganglia if compared with ADD patients.